# Machine Learning-Based Approach towards Identification of Pharmaceutical Suspensions Exploiting Speckle Pattern Images

**DOI:** 10.3390/s24206635

**Published:** 2024-10-15

**Authors:** Valentina Bello, Luca Coghe, Alessia Gerbasi, Elena Figus, Arianna Dagliati, Sabina Merlo

**Affiliations:** Department of Electrical, Computer and Biomedical Engineering, University of Pavia, 27100 Pavia, Italy; valentina.bello@unipv.it (V.B.); luca.coghe01@universitadipavia.it (L.C.); alessia.gerbasi@unipv.it (A.G.); elena.figus02@universitadipavia.it (E.F.); arianna.dagliati@unipv.it (A.D.)

**Keywords:** imaging statistics, light scattering, machine learning, optical sensing, artificial nutrition, speckle pattern imaging, turbid suspension drugs

## Abstract

Parenteral artificial nutrition (PAN) is a lifesaving medical treatment for many patients worldwide. Administration of the wrong PAN drug can lead to severe consequences on patients’ health, including death in the worst cases. Thus, their correct identification, just before injection, is of crucial importance. Since most of these drugs appear as turbid liquids, they cannot be easily discriminated simply by means of basic optical analyses. To overcome this limitation, in this work, we demonstrate that the combination of speckle pattern (SP) imaging and artificial intelligence can provide precise classifications of commercial pharmaceutical suspensions for PAN. Towards this aim, we acquired SP images of each sample and extracted several statistical parameters from them. By training two machine learning algorithms (a Random Forest and a Multi-Layer Perceptron Network), we were able to identify the drugs with accurate performances. The novelty of this work lies in the smart combination of SP imaging and machine learning for realizing an optical sensing platform. For the first time, to our knowledge, this approach is exploited to identify PAN drugs.

## 1. Introduction

### 1.1. Parenteral Artificial Nutrition

Parenteral artificial nutrition (PAN) is a medical treatment that consists of delivering liquid nutrients intravenously to those patients who are, permanently or temporarily, unable to feed themselves autonomously by assuming food and beverages by mouth. PAN is a lifesaving therapy for a large number of patients suffering from a plethora of pathologies, such as eating disorders, inflammatory bowel disease, COVID-19, and cancer [1,2,3]. Furthermore, PAN is very common among children, teenagers, and, in particular, newborns suffering from premature birth complications [4,5,6]. Some types of PAN drugs are transparent fluids constituted by a combination of water, glucose, amino acids, and electrolytes with different concentrations. Other types of PAN drugs also contain lipids; they are composed of fat micelles suspended in the surrounding liquid matrix and have a typical whitish milk-like appearance. A serious issue related to PAN therapy is represented by the high prevalence of medication human errors, as reported by the scientific literature [7]. In particular, around 75% of the errors happens during transcription, ordering, labeling, and administration of the therapy [8]. For instance, the wrong PAN mixture is often ordered through the hospital pharmacy computer system and then administered to the patient. In other cases, the label of the PAN drug given to the patient deviates from the written order, and sometimes the prescription by the caregiver is illegible, leading to misinterpretation or miscalculation [9]. Other common types of medication errors are the application of wrong labels onto the PAN mixture bags, incorrect preparation techniques, omission of essential components, or addition of incorrect ingredients. Quite often, substances that have the same appearance of PAN suspension drugs, but are completely different substances, are harmfully injected into blood vessels; in more detail, animal or breast milk, and enteral mixtures (i.e., mixtures that are intended to be administered via nasogastric tubes) are mistaken for PAN drugs. In many of these cases, the consequences on patients’ health can be dramatic and include infections, poisoning, heart and kidney failure, microembolism, allergic reactions, and even death. For example, Döring et al. reported the case of a six-week-old patient who received a 5 mL intravenous infusion of breast milk [10]. The infant immediately developed tachycardia, tachypnea, and a decrease in oxygen saturation. In [11], the case of a two-month-old infant suffering from ileal perforation and short bowel syndrome is reported, who received 25 mL of breast milk intravenously. He was treated with broad-spectrum antibiotics but died after two months because of septicemia. In another case, an enteral (i.e., for infusion in the digestive system) formula solution was administered intravenously to a 64-year-old man [12]. He developed fever, hypertension, and tachycardia, and had to be treated with antibiotics for several days. In [13], the authors report the case of a 77-year-old woman who died because of acute respiratory failure due to inadvertent intravenous infusion of enteral feed. In another situation, the wrong nutrition suspension was unintentionally administered intravenously to a woman with chronic lymphocytic leukemia [14]. The patient manifested acute multiorgan failure and septic shock. She eventually died 111 days after the infusion. Despite the remarkable number of medical errors in PAN administration, currently, there are no methods that are conventionally adopted to perform safety controls on the PAN drugs, in particular on those containing lipids and having a whitish, opaque texture, which can be easily mistaken for milk or other suspensions. Within this framework, the importance of developing innovative safety protocols and sensing devices to check the correctness of the nutrition suspension that is being administered to the patient is undeniable, and our work represents the first attempt toward this aim.

### 1.2. Speckle Pattern Imaging for Analysis of Turbid Fluids

Opto-fluidic platforms are particularly suitable for the analysis of liquids for medical applications, since optical measurements can be contactless, non-invasive, are immune to electromagnetic interference, and make use of non-ionizing radiation. However, it must be noted that, while recognition of transparent mixtures can be performed with more traditional optical sensors [15], PAN suspensions containing lipids appear as whitish fluids, similar to milk, and they cannot be investigated with the same conventional methods. Indeed, if this kind of fluid are illuminated by coherent radiation, the particles contained in the suspension act as scattering elements and light is scattered in all directions. When observed by an imaging system, diffused light is characterized by a peculiar granular texture known as a speckle pattern (SP). Hence, SP can be exploited to study turbid liquids. Traditionally, static SP imaging has been employed, for example, to study solid objects and surface roughness, to measure the thermal strain and the elastic modulus of mechanical specimen [16,17,18,19], or, more recently, to characterize the granularity of a powder surface [20]. However, the particles contained in liquid suspensions undergo Brownian motion; the SP they generate is time-varying and, thus, more challenging to investigate. In the last decade, methods based on excitation and analysis of SP have been used to examine turbid liquids that cannot be studied with optical methods traditionally applied to transparent liquids. For example, Jayanthy et al. presented laser speckle contrast analysis as a tool for measuring the concentration of static scatterers in phantom body fluids [21]. Héran et al. separately studied the p- and s-polarized SP combined with a chemometric approach to measure the absorption and scattering coefficients of turbid fluids [22]. In [23], the authors analyzed polarized SP images produced by suspensions of polystyrene microspheres by comparing the results obtained with two different experimental deployments: a light transmission setup and a backscattering configuration. Eventually, our very recent work [24] proved the suitability of the SP imaging technique to analyze real-world suspensions containing lipids, such as commercial plant-based milks. In particular, in [24], we simply analyzed dilutions obtained by adding aliquots of water staring from one single sample of pure rice milk. More recently, scientists have started exploiting Artificial Intelligence (AI) to pull out information that images of dynamic SP are full of [25]. Indeed, when dealing with dynamic SP generated by fluids, it is necessary to acquire a large amount of experimental data, extract many complex statistical features, and unveil complicated patterns to carry out reliable analyses. AI algorithms are, thus, a perfect tool to deal with this scientific challenge. For instance, Jakubczyk et al. applied convolution neural networks to SP images generated from nanoparticle suspensions [26]. After training, the algorithm was able to recognize 73 classes of particles of different materials, sizes, and concentrations. In [27], Yan et al. used a transmission configuration to collect images of the SP produced by illuminating suspensions of plastic microspheres and milk powder with a He-Ne laser; then, they used a deep learning model for automatic recognition of the samples with different particles concentrations, showing a good clustering capability. Endo et al. used a very similar configuration and applied convolutional neural networks to discriminate between the size and concentrations of microplastics [28]. These very few preliminary works are surely interesting, however they are all based on Convolutional Neural Networks (CNNs), which directly take SP images as input and work as “black box” models at the expense of their interpretability and explainability. Hence, it is difficult to understand how they make decisions based on the features extracted from images. Moreover, the works reported in [26,27,28] only consider and measure suspensions of plastic and metal nanoparticles with well-known geometry and dimension, prepared ad hoc in the laboratory. They do not deal with real-world suspensions, such as PAN drugs or other types of commercial/biological fluids, which are much more complex and less controlled in term of composition and physical features.

### 1.3. Work Motivations

In this work, we have designed and implemented a sensing platform based on combination of SP imaging and AI for automatic recognition of real, commercial pharmaceutical suspensions employed in PAN. The goal of our work is, thus, to compensate for the existing lack of sensing devices for the identification of turbid PAN drugs and address the issue of medical error in artificial nutrition. We have exploited a simple experimental configuration based on a low-cost semiconductor laser diode and a CMOS digital camera to acquire sequences of SP images of six commercial mixtures used for PAN. Then, SP data were processed, and many statistical parameters were extracted from every frame. Eventually, these statistical features were used as input to a machine learning (ML)-based pipeline for identification of the pharmaceutical suspensions. We preferred the use of ML models (a Random Forest and a Multi-Layer Perceptron Network) to more widespread CNNs in view of better interpretability of the automatic decision process and to deeply understand the contribution of each statistical feature. Moreover, in our work, we have also tried to provide an explanation on how the statistical features affects the model’s predictions. In more detail, Section 2.1 reports the description of the suspension drugs tested in this work. In Section 2.2, the experimental configuration is presented. The procedure used for data collection and feature extraction is described in Section 2.3, and Section 2.4 reports the ML pipeline that we followed. Eventually, the results of the work and the discussion and conclusions are reported in Section 3 and Section 4, respectively.

## 2. Materials and Methods

### 2.1. Suspension PAN Drugs

**Figure 1:**
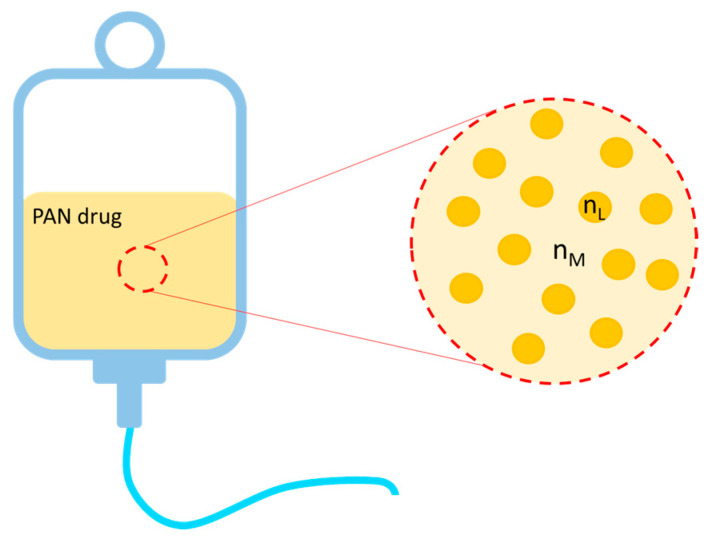
Schematic representation of the microscopic structure of PAN suspensions. Lipid micelles are surrounded by the liquid matrix with different refractive indexes, thus acting as scattering particles.

PAN drugs are multicomponent medications, and they are among the most complex therapies of modern medicine [29]. In this work, we have investigated six commercial PAN suspensions produced by Baxter (Deerfield, IL, USA) with the commercial names OLIMEL N5E, OLIMEL N7E, OLIMEL N12E, OLIMEL N4E, FINOMEL Periferico, and NUMETA G13E. Their chemical composition is reported in Table 1. Each PAN suspension was contained in a bag with three compartments (one containing the glucose solution, one containing the amino acid solution, and one containing the lipid emulsion) divided by two plastic membranes which needed to be broken to constitute the PAN suspension. After mixing, the constituted PAN drugs could be stored in their bags inside the refrigerator for up to 7 days. In the resulting liquid, glucose, electrolytes, and some types of amino acids were dissolved in water and, together, they constituted the liquid matrix surrounding the lipid micelles suspended in the mixture (Figure 1). The fat micelles were spherical particles with sizes approximately between 100 and 500 nm [30]. Moreover, the micelles were derived from olive and soybean oil and/or fish oils; in particular, FINOMEL was the only drug investigated here that also contained fish oil, while the other suspensions were composed of vegetable oils only. The oil micelles had a refractive index (RI) *n_L_* of about 1.450–1.485 RIU [31,32], while the RI of the surrounding liquid matrix *n_M_* was mainly influenced by the content of water and glucose. According to theoretical calculations on *n_M_*, we can state that (for the PAN drugs we investigated) *n_M_* varied in the range of 1.349–1.361 RIU, and it was always smaller than *n_L_*. Hence, lipids act as scattering particles inside the fluid, and the amount of scattered light depends both on the concentration of lipids and on the difference Δ*n* = |*n*_L_ − *n_M_*|.

### 2.2. Instrumental Configuration for Speckle Pattern Imaging

**Figure 2:**
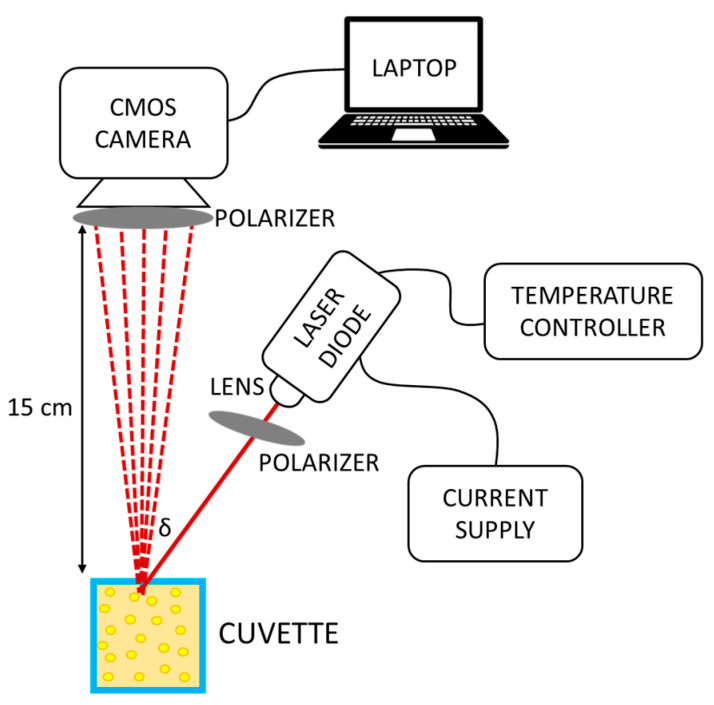
Schematic of the instrumental configuration for generation of speckle pattern and acquisition of speckle pattern images.

The instrumental configuration used to carry out experimental measurements on the pharmaceutical suspensions is reported in Figure 2. A semiconductor laser diode (L658P040, Thorlabs, Newton, NJ, USA) was used as coherent light source. It emitted a maximum optical power of 40 mW with a center emission wavelength *λ* of 658 nm and a linewidth Δ*λ* of 0.2 nm. The coherence length, which was calculated as *L_C_* ≈ *λ*/(Δ*λ*), was then equal to 2.2 mm. From the literature, it is known that the main effect of using optical sources with partial coherence to excite SP is degradation of the image contrast [33]. According to the literature, however, this effect does not seem to be significant for laser diodes with relatively long (>100 μm) coherence length, as in our case. It was powered through a current driver (LDC500, Thorlabs, Newton, NJ, USA) and connected to a temperature controller (PRO800, Thorlabs, Newton, NJ, USA) for thermal stabilization to ensure the stability of the emitted optical spectrum. An aspheric lens (C230260P-B, Thorlabs, Newton, NJ, USA) was used to focus the radiation onto the surface of a transparent plastic cuvette (volume of 4.5 mL, dimensions of 10 mm × 10 mm × 5 cm) containing the drug sample test at an angle of *δ* ≈ 30°. Since semiconductor lasers emit elliptically polarized light, a linear polarizer (LPVISE100-A, Thorlabs, Newton, NJ, USA) was positioned in front of the lens to select only the polarization component along the main axis of the ellipse. Images and videos of the SP generated by the scattering fluids were acquired using a monochrome CMOS digital camera (Alvium 1800 U-158m, Allied Vision, Stadtroda, Germany) located in front of the cuvette, on the same side of the laser. The selected orientation of the camera with respect to the laser prevented the specular reflection from the cuvette wall from reaching and saturating the CMOS sensor. The camera sensor had a size of 5.02 mm × 3.75 mm and a total number of pixels of 1456 × 1088; the dimension of each pixel was 3.45 µm × 3.45 µm. A second linear polarizer, identical to the other, was placed in front of the CMOS sensor. The camera had a USB connection to a laptop that allowed for control of the acquisition parameters by means of proprietary software (Vimba, Allied Vision, Stadtroda, Germany). Processing of the collected videos and statistical investigation were carried out by a custom written MATLAB 2022b script, as described in the following section.

### 2.3. Data Collection and Feature Extraction

**Figure 3:**
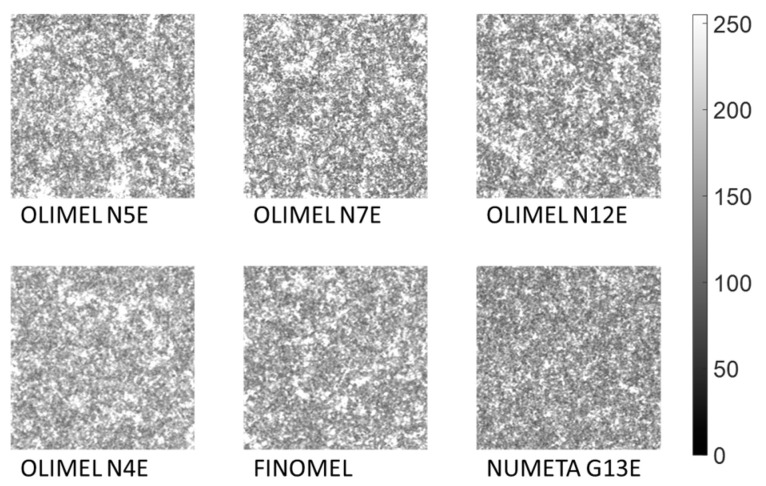
Example of cropped SP frame with 224 × 224 pixels acquired for each PAN suspension. The scale bar represents the gray level intensity with values ranging from 0 (black) to 255 (white).

We used the experimental setup of Figure 2 to collect SP videos made up of 100 frames for every PAN drug. Four samples of each PAN drug (taken from the same drug bag) were tested: two samples were tested on the day of suspension preparation; the other two samples were tested after two days. For each sample, three videos were collected (each composed of 100 SP frames). Thus, a total of 1200 SP images were obtained for each one of the six PAN drugs, resulting in a dataset of a total of 7200 SP images. All samples were tested at room temperature (25 °C). All SP images were captured using identical instrumental settings and experimental conditions: the laser current was set to 80 mA (corresponding to an emitted optical power of roughly 38 mW), the framerate to 83 fps, and the exposure time to 602 µs. Videos were acquired in raw format with a depth of 8 bit, leading to 256 gray levels, ranging from 0 (corresponding to black) to 255 (corresponding to white). We created a dataset consisting of 7200 SP frames, containing 1200 images for each one of the six PAN drugs under investigation. Data were pre-processed in the MATLAB 2022b environment: each SP frame was converted into a grayscale level matrix, and we employed a center-cropping technique to reduce the image dimension from 1456 × 1088 pixels to 224 × 224 pixels. This choice was motivated by the remarkable computational resources required for processing very large images and by the choice to avoid resizing with interpolation, since that could distort the SP. The SP was uniform across the entire image, and there were no significant variations between the center and other regions of the image. Working with the full-size images would have been both computationally impractical and unnecessary, given the uniformity of the pattern. To confirm that the cropped image was a representative portion of the image, we carried out an analysis of the gray level distribution: in our specific case, the grayscale intensity distribution was consistently uniform across the entire image. We also applied hypothesis tests, which confirmed that there were no statistically significant differences between full-size images and cropped images. Hence, we could safely assume that even a portion of the image adequately represented the statistical information contained within the entire image. The 224 × 224 image size is also suitable for future applications involving pre-trained deep learning models, where this dimension is a common standard input size. This would allow for direct comparison between our current results, which rely on hand-crafted feature extraction, and potential deep learning-based approaches. As an example, Figure 3 shows a sample cropped image for each drug included in this study.

It is important to note that the six SP frames reported in Figure 3 exhibit a high degree of visual similarity, making it challenging to distinguish the drugs through visual inspection alone. This observation highlights the necessity and appeal of using ML algorithms to accurately differentiate between the drugs based on their underlying texture characteristics. Subsequently, we partitioned our image dataset into training (80%, corresponding to 5760 SP images) and test (20%, corresponding to 1440 SP images) sets. Following this step, we normalized the gray level intensities. We adopted this strategy because the formation of SP could also be influenced by acquisition conditions not related to the sample characteristics. These conditions may include factors such as temperature variations, potential micro-scratches on the cuvette walls, and minor fluctuations in laser optical power, even under a constant supply current. To ensure comparability between images acquired from the same sample but at different times, it was imperative to account for these potential sources of variability. Since, for each sample, we conducted four separate experiments on two distinct days, in order to take into account the fact that measurements could have been performed in slightly different experimental conditions, we performed intensity normalization independently for each drug using the training set images. Finally, we rescaled all values within the range of 0 to 255 for each image and extracted a comprehensive set of 129 statistical features. Their selection was guided by a thorough review of the literature [34]. In more detail, we extracted statistical parameters either directly from the SP images or as first- and second-order statistical measures derived from Gray-Level Co-occurrence Matrices (GLCMs) and Gray-Level Run-Length Matrices (GLRLMs). Specifically, we computed direct statistics such as intensity, standard deviation, variance, kurtosis, and skewness for each image. Additionally, for each SP image, we calculated its GLCM, which records the frequency of specific pairs of pixel values appearing together in the image, and its GLRLM, which quantifies the length of gray level runs, defined as consecutive pixels with specific gray level values, thereby capturing texture and patterns within the image. The GLCM represents the frequency of occurrence of a pair of grey levels at a specified distance *d* apart and along a specified direction at angle *θ*. If the grayscale matrix *I* representing the SP image has dimensions *M* × *N* and contains *K* gray levels, the GLCM is a *K* × *K* matrix. The (*i*, *j*)_th_ element of the GLCM represents the number of times a pixel with intensity *i* is neighbor to a pixel with intensity *j,* and is calculated as

(1) $$GLCM_{\theta }\left(i,j\right)=\sum_{n=1}^{N}\sum_{M=1}^{M}\left\{\begin{array}{l} 1,\;if\;I\left(m,n\right)=i\;and\;I(m+d\;cos\left(\theta \right),\;n+d\;sin\left(\theta \right))=j\\ 0,\;otherwise \end{array}\right.$$

where *d* can be varied from 1 to the size of the SP image. In our work, we considered only *d* = 1 [34]. In the GLRLM, its (*i*, *j*)_th_ element describes the number of runs with gray level *i* and length *j* occurring in the image along a specified direction *θ*. Both GLCM and GLRLM computations were performed considering the four possible directions (*θ* = 0°, 45°, 90°, 135°) of translation along the image, and multiple statistical measures were derived for each matrix. An exhaustive list of the computed features is presented in the Supplementary Materials for reference.

### 2.4. Machine Learning Pipeline

**Figure 4:**
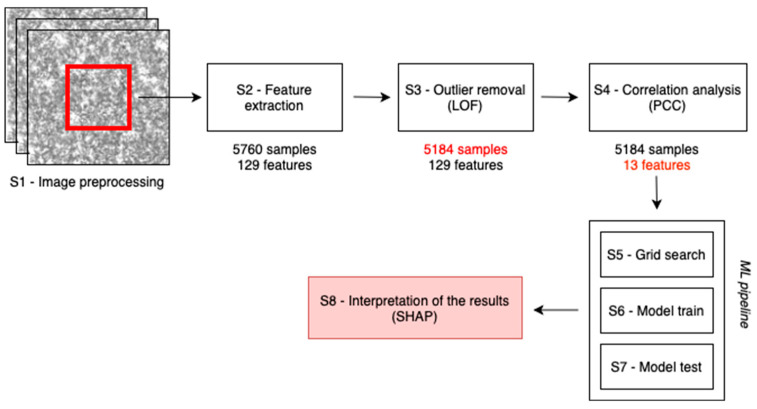
Overview of the main pipeline steps. The process begins with image cropping and preprocessing (step S1), followed by feature extraction (step S2), outlier removal (step S3), and correlation analysis (step S4). Feature selection is based on removing redundant features after the correlation analysis. The machine learning pipeline (steps S5–S7) includes grid search for hyperparameter optimization, model training, and model testing. Finally, the results are interpreted using SHAP (step S8) to identify the most relevant predictors.

We developed a fully automated ML pipeline based on statistical features extracted from SP images. The described pipeline is schematically represented in Figure 4, with each step numbered form S1 to S8. Our primary objective was to accurately identify the six PAN drugs from imaging statistical features.

First, we cropped the original full-sized images (Figure 4, step S1) and extracted the 129 features (Figure 4, step S2) as previously described. Then, we employed the local outlier factor (LOF) algorithm [35] to detect outliers within the training dataset (Figure 4, step S3). LOF is an unsupervised anomaly detection method that calculates the local density deviation of a data point in relation to its neighboring data points. In our case, we used the Euclidean distance with a default neighbor count of 20, which has proven effective in most scenarios. Any data points with significantly lower density compared to their neighbors were considered as outliers. In this step, we removed 10% of the samples with the lowest LOF scores.

After outlier removal, we proceeded to reduce the dimensionality of the feature set by eliminating highly collinear variables within the training data (Figure 4, step S4). This step helped to alleviate computational overhead by excluding variables that essentially conveyed the same information. To achieve this aim, we calculated the Pearson correlation coefficient (PCC) [36] and randomly removed one of the variables from pairs with an absolute PCC value ≥ 0.9. The feature selection step greatly aided in eliminating redundant information and simplifying the training and interpretation of the final classification models.

We then compared two different classification models: a Random Forest (RF) [37] and a Multi-Layer Perceptron Network (MLP) [38]. We opted for these two models due to their strengths and suitability for this specific task. Random Forest, a tree-based ensemble method, is known for its robustness against overfitting and its ability to handle high-dimensional datasets effectively. MLP, a type of artificial neural network, is able to capture complex nonlinear relationships within the data, thus learning intricate patterns and representations, which can be critical when dealing with SP image-based data. The parameters of these models were tuned through a grid search (Figure 4, step S5) conducted in conjunction with a 10-fold cross-validation on the training set. We trained the models on the training set (Figure 4, step S6) and then validated them on the test set (Figure 4, step S7). The performance of the final models was assessed in terms of accuracy, sensitivity, specificity, precision, and the area under the ROC (Receiver Operating Characteristic) curve (AUC) using the test set.

An essential aspect of our work consists of identifying features that can effectively be exploited as effective parameters to distinguish the different drugs. Consequently, the interpretability of the implemented model is of utmost importance. Currently, the state-of-the-art model-agnostic explainability method for tabular data is SHapley Additive exPlanations (SHAP) [39], which we exploited in our work (Figure 4, step S8). This method for estimating SHAP values has been shown to align better with human intuition compared to alternative techniques. The idea behind Shapley values is rooted in game theory; the role of each feature in determining the ultimate prediction is akin to the contribution of individual players in a cooperative game, such as football, for instance. This allows us to interpret the role of each feature to the final classification, determining the most influencing parameters. We present both the quantitative and qualitative results of each analysis step in the following section for thorough discussion and analysis.

## 3. Results

**Figure 5:**
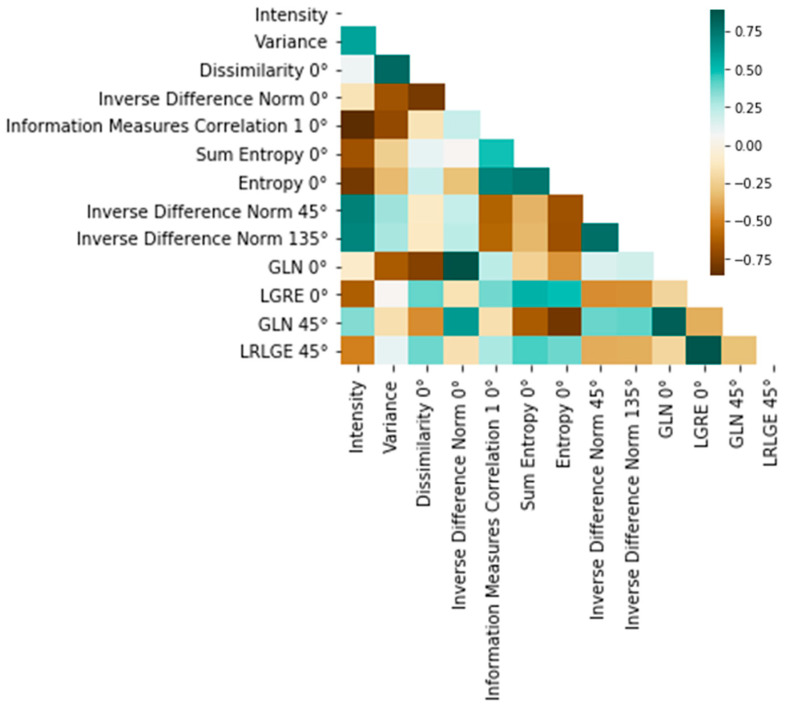
Correlation heatmap showing the PCC for the features left after feature selection, which was carried out on the basis of the study of the correlation strength.

After the preprocessing operation of image cropping and normalization (Figure 4, step S1), followed by the feature extraction procedure (Figure 4, step S2) described in the previous sections, the subsequent operation involved outlier removal (Figure 4, step S3), reducing the training dataset from 5760 to 5184 samples. Subsequently, we computed the correlation heatmap and, deleting collinear variables, we obtained a significant reduction in the feature set size, from 129 to 13 (Figure 4, step S4). The remaining features with PCC < 0.9 are reported in the correlation heatmap of Figure 5.

This reduction can be expected, as many of the computed characteristics were extracted by the GLCM and GLRLM matrices computed along multiple directions and inherently correlated by design, since SP does not develop along a preferential direction; rather, it is randomly distributed in the observation plane. The best parameters resulting from the grid-search procedure (Figure 4, step S5) are provided in Table 2 and Table 3 for the RF and MLP models, respectively.

**Figure 6:**
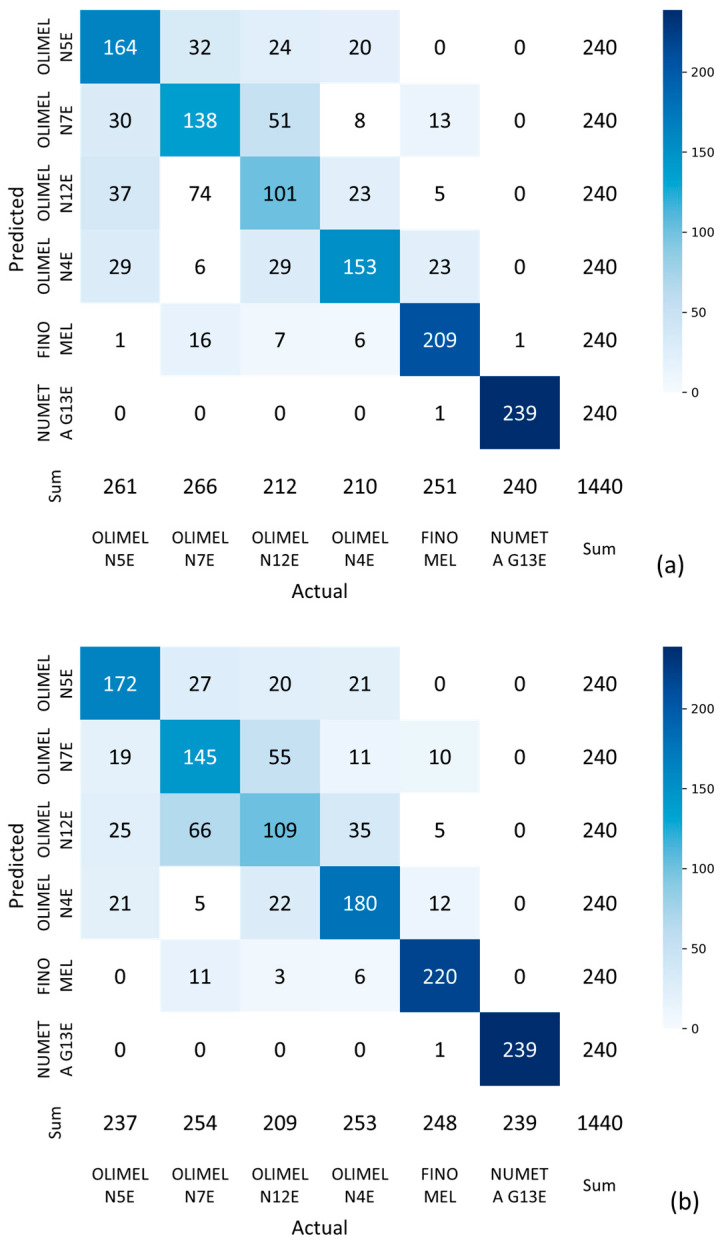
Confusion matrices obtained by applying the RF model (**a**) and the MLP model (**b**) for drug classification. The vertical bar represents the number of classified instances, with color ranging from white (representing the 0 value) to dark blue (representing the value of 240, corresponding to the maximum number of instances for every PAN drug considered in the test of the model).

The selected models were trained using the optimal parameters on the training set (Figure 4, step S6) and finally tested on the independent test set (Figure 4, step S7). For both models, the confusion matrices are reported in Figure 6; from them, we extracted the summary metrics representing the average performance in the multiclassification task with 95% confidence intervals (CI) on the test set (Table 4). Notably, the MLP was the top-performing model, achieving an overall AUC of 0.94 [95% CI: 0.93, 0.95].

From now onwards, we are focusing our analysis on the MLP model, since it has been shown to have the best performance. Upon examining its confusion matrix (Figure 6b), it became evident that NUMETA G13E was perfectly distinguished from all the other drugs in almost 100% of the cases, and FINOMEL was very often identified correctly (in 220 cases over 240). Indeed, NUMETA G13E is quite different in composition form the other drugs, since it has a very low lipid content, but a high glucose concentration (as reported in Table 1). With regard to FINOMEL, its composition is similar (in term of concentration of nutrients) to that of OLIMEL N4E; however, FINOMEL is the only PAN drug in our study containing fish oil together with plant-based oils: this feature could make it easier to be distinguished. On the other hand, most errors occurred when the model attempted to distinguish between OLIMEL N12E and OLIMEL N7E.

**Figure 7:**
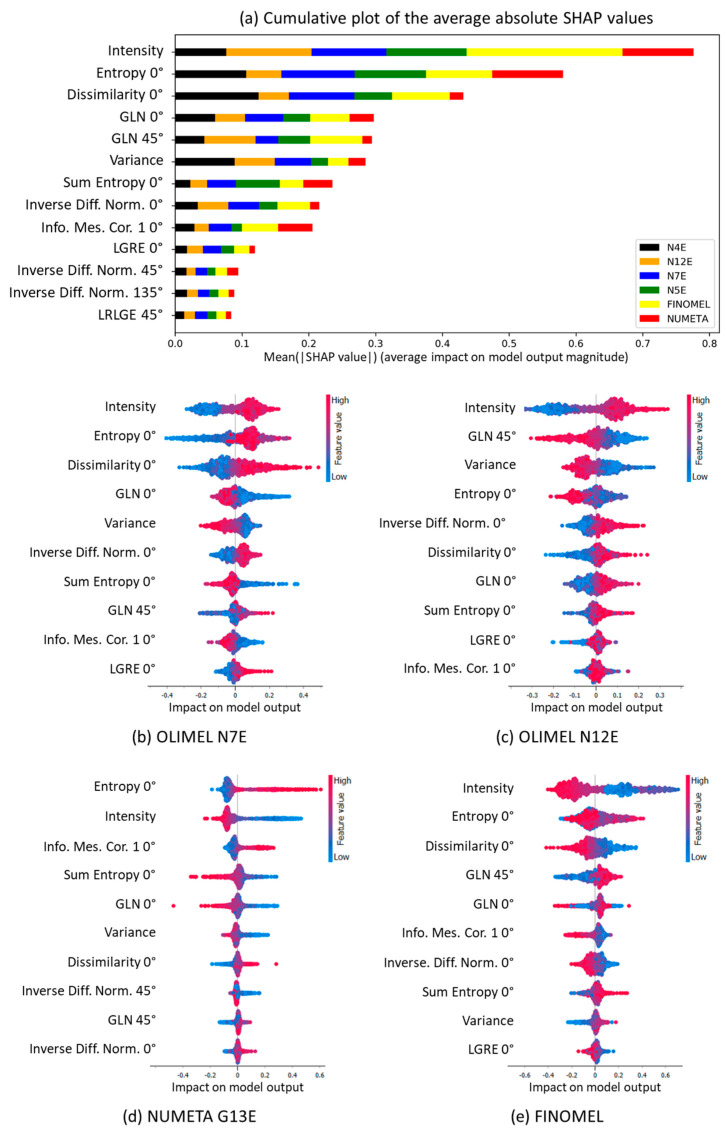
SHAP plots obtained with the MLP model. (**a**) Cumulative plot of the average absolute SHAP values for all the features included in the model. Beeswarm SHAP plots for OLIMEL N7E (**b**), OLIMEL N12E (**c**), NUMETA G13E (**d**), and FINOMEL (**e**) drugs.

As a final step of our analysis pipeline (Figure 4, step S8), to understand the role of each feature in the classification algorithm, we computed and analyzed the SHAP plots of Figure 7, obtained from the MLP model. Figure 7a provides a cumulative plot representing the average absolute SHAP values for all the features included in the model. This representation is useful to understand the average impact of each feature on model outcome. We can observe that the most influencing features are intensity, variance, and dissimilarity at 0°. Intensity is the average gray level value within the image and refers to the average brightness or luminance of the SP image. High-intensity values indicate a higher density of bright speckle grains in the image and are related to a higher concentration of scattering elements (i.e., lipids). Conversely, low-intensity values might suggest a lower scatterer concentration. However, it must be considered that intensity is also strongly affected by the parameter Δ*n* that was defined in Section 2.1, “Suspension Drugs for Parenteral Nutrition”, as the difference between the micelles RI and the liquid matrix RI. Hence, PAN drugs with higher contents of glucose and amino acids will have a higher *n_M_* and, thus, a smaller Δ*n*, which can be a motivation for a lower intensity of the backscattered light. To sum up, the intensity is determined by the combined effect of the lipid concentration and pixel values within the SP image. It evaluates how much the pixel values deviate from an ideal uniform distribution. High entropy values may indicate a complex and heterogeneous distribution of features in the speckle pattern, with a wide range of pixel values. Low entropy values, on the other hand, suggest a more uniform and consistent distribution of features, signifying that the speckles are more similar in their gray-level values. Eventually, dissimilarity at 0° measures the dissimilarity between gray-level values in the SP image along the 0° orientation. High dissimilarity values suggest that there are significant differences between neighboring pixels, which can be indicative of fine details or structural variations in the sample. Conversely, a low dissimilarity value implies a more uniform texture or similarity between neighboring pixels, indicating that the speckles are more similar to their immediate neighbors. These features have a substantial impact on the model’s predictions and its ability to distinguish among different drugs based on SP images. Understanding their significance can aid in the further interpretation of model performance. For instance, if intensity is a dominant feature, it could imply that differences in the average brightness play a crucial role in drug discrimination. Meanwhile, the influence of entropy and dissimilarity at 0° suggests that the complexity and fine-grained textural details in the SP are also vital for classification.

To further understand how these characteristics influence the model prediction for single drugs, we provide the beeswarm plots shown in Figure 7b–e. Beeswarm plots arrange features by the sum of their SHAP value magnitudes across all samples, revealing the impact of each individual feature on the *x*-axis. The original feature values are represented by colors ranging from blue (low values) to red (high values). By analyzing the beeswarm plots for OLIMEL N7E and OLIMEL N12E (Figure 7b,c), we observe a consistent trend. In both cases, intensity appears to be the most influential feature, with higher values (indicated in red) associated with positive SHAP values. Consequently, these high intensity values push the model prediction towards both classes, making it more difficult for the algorithm to succeed in the distinction.

**Figure 8:**
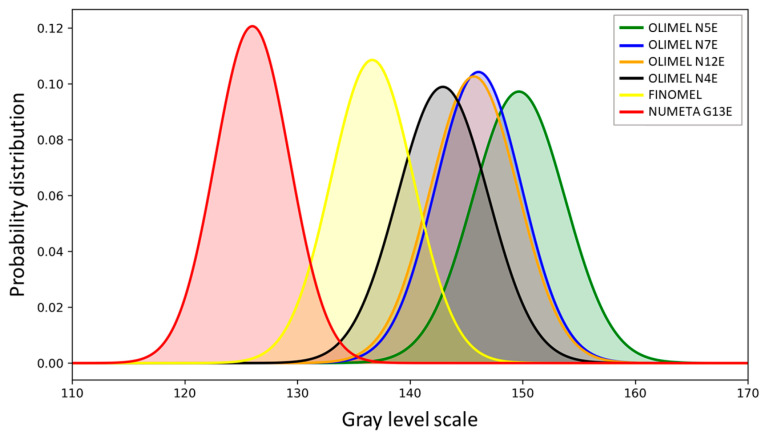
Probability distribution of the gray level intensity of each PAN drug (calculated after standardization). The distributions of OLIMEL N7E and OLIMEL N12E are highly overlapped.

Given that intensity is, overall, the most informative feature identified by our model, we further investigated how well the model can distinguish between OLIMEL N12 and OLIMEL N7. To do so, we conducted an examination of the probability distributions of the SP gray level values for each drug, as illustrated in Figure 8. We employed an ANOVA test to statistically compare these distributions, using a significance threshold of 0.01. The ANOVA test result, with an F-statistic of 4627.77 and a *p*-value ≤ 0.001, provides strong evidence against the null hypothesis, which asserts that there is no significant difference in the probability distributions of the SP gray level values between the different drug groups. In other words, given the very low *p*-value obtained with the ANOVA test, we can confirm that there is a statistically significant difference between the gray-level distributions.

To better inspect which pairs of groups were different from each other, we performed Tukey’s HSD (Honestly Significant Difference) Pairwise Group Comparisons test, testing the null hypothesis that the distributions underlying the pair of samples had the same mean. The results of this test are reported in the Supplementary Materials file, and they offer insights into the specific intergroup differences. It became evident that, once again, using a significance threshold of 0.01, we could consistently reject the null hypothesis (*p*-value < 0.001) in all cases except for the OLIMEL N7E-OLIMEL N12E pair. This finding is consistent with the higher error rate of the model in recognizing these two drugs. Although intensity remained the most discriminative feature when considering the overall multiclass problem, the similarity in the probability distribution of intensity values for OLIMEL N7E and OLIMEL N12E contributed to the elevated error rate. By examining the beeswarm plot of NUMETA G13E (the drug that is more easily identified) in Figure 7d, it becomes apparent that SP images associated with this drug exhibit specific characteristics. These characteristics include very high entropy at 0° values, indicating a significant degree of randomness or disorder of the gray level of the pixels at an orientation of 0°. Additionally, these images have very low intensity values, indicating low illumination. Moreover, they demonstrate a very high information measure correlation at 0°. This means that the gray-level values of the pixels in these images are strongly related to each other, and changes in one gray-level pixel are positively associated with changes in another gray-level pixel, indicating a certain degree of regularity or predictability in the pixel patterns. Therefore, high entropy at 0°, low intensity, and a high positive information measure correlation at 0° are the top three features associated with positive SHAP values. This outcome suggests that they play a pivotal role in the model’s accurate identification of NUMETA G13E. Finally, if we observe Figure 7e, we can see that, in SP images generated by FINOMEL samples, areas with low brightness are associated with positive model influence, suggesting that regions with reduced illumination play a crucial role in its identification. On the other hand, extreme values of entropy at 0°, either very high or very low, have a negative impact on the model’s prediction, indicating that intermediate values, representing a balance between randomness and order, are more associated with this drug. Furthermore, very low values of dissimilarity at 0°, signifying high similarity among neighboring pixels, are linked to a positive model influence, highlighting the particularity of consistent and uniform textures found in FINOMEL images.

## 4. Discussion and Conclusions

In this work, we successfully realized a smart sensing configuration by combining SP imaging with an AI analysis for the identification of commercial pharmaceutical suspensions used in PAN, using the statistical features of SP images. To the best of our knowledge, this is the first time an ML pipeline has been exploited to analyze SP images for the classification of complex suspension drugs. After excitation and acquisition of SP with a simple and relatively low-cost optoelectronic sensing setup, as well as extraction of the statistical parameters, we applied and compared an RF model and an MLP model. MLP has proven to be the better-performing model. When applied to the test set, it was able to recognize four of the six drugs with an accuracy of over 70% (in 99.6%, 91.7%, 75%, and 71.7% of cases, respectively). On the other hand, the MLP seemed to fail to correctly identify OLIMEL N12E and OLIMEL N7E (with accuracies of 60.4% and 45.4%, respectively).

While our initial goal was to approach this problem as a multiclass classification task and to identify the most informative features for distinguishing among all six drugs, it became evident that, for OLIMEL N12E and OLIMEL N7E, these features may not be optimal predictors. Moreover, the difficulty in distinguishing OLIMEL N12E and OLIMEL N7E stemmed from the fact that these two drugs exhibit very similar visual characteristics, leading to the model’s lower accuracy for these cases. In contrast, the remaining drugs were more distinct in their SP image features, enabling the model to achieve much better results. Thus, the overall performance for most of the drugs was promising, but further refinement may be needed to address the challenges associated with the OLIMEL formulations. It is important to note that this work represents the first attempt to apply ML to SP images for drug identification, making it challenging to directly compare our results with other benchmarks in the literature. Therefore, it could be valuable to explore whether developing a dedicated classification algorithm specifically tailored for these two drugs would improve the performance. This approach could potentially uncover additional discriminative features that are more effective in differentiating between these similar formulations. Moreover, for this study, we focused exclusively on imaging features to assess the informativeness of SP image patterns. However, future work could consider incorporating additional information, such as the drug compositions provided in Table 1. Including these chemical properties in the feature set could enhance the model’s ability to differentiate between drugs, leading to SP images with similar visual characteristics. Since we already have access to the drug composition data, incorporating these factors as additional predictors would be a straightforward extension of our approach.

While we approached this work as a multiclassification problem, with the aim to find the best parameters able to distinguish among all the examined drugs, we also discovered that, for at least two drugs (OLIMEL N12E and OLIMEL N7E), these characteristics may be suboptimal. In this context, it would be valuable to explore whether an ad hoc classification algorithm, developed downstream from the one we proposed, could reveal better discriminative features specific for the OLIMEL drugs. For this experiment, we decided to rely only on imaging features to evaluate the informativeness of SP image patterns. As a future development, it could be interesting to retrain the model by adding the information related to the drug compositions to the feature set (as reported in Table 1).

In conclusion, to the best of our knowledge, in our work, for the first time, SP imaging is combined with ML models to classify real-world PAN drugs constituted by a suspension of lipids in a solution of water, glucose, and amino acids. While a few other works reported in the literature rely on “black-box” CNN models, our research is based on the extraction of statistical features and the use of SHAP values, which allowed us to obtain better insight into the algorithm’s explainability. Future works could also explore the utilization of state-of-the-art deep learning models, such as, for example, Vision Transformers. These techniques offer the advantage of fully automated feature extraction, although it may come at the cost of reduced interpretability if compared to the approach we have proposed. In addition, these methods have the potential to extract more generalized characteristics from the data, which could be valuable for broader applications and datasets. This research path could lead to a deeper understanding of the data’s underlying patterns and contribute to the enhancement of classification performance in a wider context.

## Figures and Tables

**Table 1:** Composition of the commercial suspension drugs for parenteral nutrition investigated in this work.

Drug	Lipids (g/L)	Amino Acids (g/L)	Glucose (g/L)	Calories (kcal/L)
OLIMEL N5E	40.00	32.93	115.00	990
OLIMEL N7E	40.00	44.27	140.00	1140
OLIMEL N12E	35.07	76.00	73.38	954
OLIMEL N4E	30.00	25.30	75.00	700
FINOMEL	28.20	31.51	70.69	1091
NUMETA G13E	25.00	29.47	133.33	910

**Table 2:** Best parameters for the RF model resulting from the grid-search procedure.

Split Criterion	Max. Tree Depth	# of Features Considered for Best Split	Min. # of Samples for Leaf Definition	Min. # of Samples for Node Definition	# of Trees
entropy	10	4	1	5	250

**Table 3:** Best parameters for the MLP model resulting from the grid-search procedure.

Activation Function	Hidden Layer Size	Max. # of Iterations	Batch Size	Learning Rate	Solver	Loss Function
hyperbolic tangent	(100, 25)	200	200	0.001	Adam algorithm	Categorical cross- entropy

**Table 4:** Summary metrics representing the average performance in the multiclassification task with 95% confidence intervals calculated by the test for both RF and MLP models.

Model	Accuracy	Sensitivity	Specificity	Precision	AUC
**RF**	0.697 [0.673, 0.720]	0.697 [0.673, 0.720]	0.924 [0.909, 0.934]	0. 0.697 [0.673, 0.720]	0.931 [0.917, 0.943]
**MLP**	0.740 [0.717, 0.762]	0.740 [0.717, 0.762]	0.937 [0.923, 0.948]	0.736 [0.713, 0.758]	0.941 [0.927, 0.952]

## Data Availability

The data presented in this study are available on request from the corresponding author.

## Supplementary Materials

The following supporting information can be downloaded at: https://www.mdpi.com/article/10.3390/s24206635/s1. Table S1: List of statistical features extracted directly from the SP images; Table S2: List of statistical features extracted from the GLCM; Table S3: List of statistical features extracted from the GLRLM; Table S4: Results of Tukey test; Figure S1: Boxplot representing the mean value of the gray-level intensity, the 25th and 75th percentile and the outliers for each PAN drug; Figure S2: SHAP values related to FINOMEL (extracted after application of the MLP model).; Figure S3: SHAP values related to OLIMEL N4E (extracted after application of the MLP model); Figure S4: SHAP values related to OLIMEL N5E (extracted after application of the MLP model); Figure S5: SHAP values related to OLIMEL N7E (extracted after application of the MLP model); Figure S6: SHAP values related to OLIMEL N12E (extracted after application of the MLP model);Figure S7: SHAP values related to NUMETA G13E (extracted after application of the MLP model).
