# Supplementary material for: Machine Learning-Based Approach towards Identification of Pharmaceutical Suspensions Exploiting Speckle Pattern Images
Source: Sensors (Basel). 2024 Oct 15;24(20):6635. doi: 10.3390/s24206635 (PMC11511328; doi:10.3390/s24206635)
Supplement: Supplementary file 1 [file sensors-24-06635-s001.zip › sensors-3068985-supplementary.pdf]

Supplementary Material to the article:

Machine Learning-Based Approach towards Identification of Pharmaceutical Suspensions Exploiting Speckle Pattern Images

Table S1 List of statistical features extracted directly from the SP images

We here list the statistical parameters extracted directly form the gray level matrices representing the SP images, represented by the gray-scale matrix *I* with dimension *M* x *N*. In the formulas reported in the following table, *i* and *j* represent the pixel coordinates in the SP image and they vary between 1 and *M* and 1 and *N*, respectively.

Statistical feature	Mathematical expression
Intensity (average intensity)	$\hat{I} = mean = \sum_{i=1}^{M}\sum_{j=1}^{N}\frac{I(i,j)}{M \cdot N}$
Standard deviation	$std = \sqrt{\frac{1}{M \cdot N}\sum_{i=1}^{M}\sum_{j=1}^{N}\lvert I(i,j) - mean\rvert^2}$
Variance	$Var = std^2$
Kurtosis	$Kur = \frac{\frac{1}{M \cdot N}\sum_{i=1}^{M}\sum_{j=1}^{N}(I(i,j) - mean)^4}{(\frac{1}{M-1}\sum_{i=1}^{M}\sum_{j=1}^{N}(I(i,j) - mean)^2)^2}$
Skewness	$Ske = \frac{\frac{1}{M \cdot N}\sum_{i=1}^{M}\sum_{j=1}^{N}(I(i,j) - mean)^3}{(\frac{1}{M-1}\sum_{i=1}^{M}\sum_{j=1}^{N}(I(i,j) - mean)^2)^{\frac{3}{2}}}$

Table S2 List of statistical features extracted from the GLCM

We here list the statistical parameters extracted from the GLCMs (Grey-Level Co-Occurrence Matrices) calculated from the SP images. In the formulas reported in the following table, *K* represent the gray level (varying from 0 to 255); (*i*, *j*) represent the element with coordinates *i* and *j* in the GLCM matrix.

Statistical feature	Mathematical expression
Energy	$ENR = \sum_{i=1}^{K}\sum_{j=1}^{K} GLCM(i,j)^2$
Homogeneity	$Hom = \sum_{i=1}^{K}\sum_{j=1}^{K} \frac{GLCM(i,j)}{1+\lvert i-j\rvert}$
Contrast	$Con = \sum_{i=1}^{K}\sum_{j=1}^{K} GLCM(i,j) \cdot \lvert i-j\rvert^2$
Maximum probability	$max = \max\,(GLCM(i,j))$
Difference entropy	$DENT = -\sum_{i=1}^{K} GLCM_{x-y}(i) \cdot \log_2 GLCM_{x-y}(i)$ where $GLCM_{x-y}(k) = \sum_{i=1}^{K}\sum_{j=1}^{K} \delta_{\lvert i-j\rvert,k} GLCM(i,j)$ with k = 0, 1, …, K-1
Correlation	$Corr = \frac{1}{\sigma_x \cdot \sigma_y}\sum_{i=1}^{K}\sum_{j=1}^{K}(i \cdot j) \cdot GLCM(i,j) - \mu_x \cdot \mu_y$ where $\mu_x = \sum_{i=1}^{K}\sum_{j=1}^{K} iGLCM(i,j)$ $\mu_y = \sum_{i=1}^{K}\sum_{j=1}^{K} jGLCM(i,j)$

	$$\sigma_x = \left[\sum_{i=1}^{K}\sum_{j=1}^{K}(i-\mu_x)^2 GLCM(i,j)\right]^{\frac{1}{2}}$$ $$\sigma_y = \left[\sum_{i=1}^{K}\sum_{j=1}^{K}\left(i-\mu_y\right)^2 GLCM(i,j)\right]^{\frac{1}{2}}$$
Cluster	$$Clu = \sum_{i=1}^{K}\sum_{j=1}^{K}(i+j-\mu_x-\mu_y)^3 \cdot GLCM(i,j)$$ where $$\mu_x = \sum_{i=1}^{K}\sum_{j=1}^{K} iGLCM(i,j)$$ $$\mu_y = \sum_{i=1}^{K}\sum_{j=1}^{K} jGLCM(i,j)$$
Cluster prominence	$$CluP = \sum_{i=1}^{K}\sum_{j=1}^{K}\left(i+j-\mu_x-\mu_y\right)^4 \cdot GLCM(i,j)$$ where $$\mu_x = \sum_{i=1}^{K}\sum_{j=1}^{K} iGLCM(i,j)$$ $$\mu_y = \sum_{i=1}^{K}\sum_{j=1}^{K} jGLCM(i,j)$$
Sum average	$$Sum_{ave} = \sum_{i=2}^{2K} i \cdot GLCM_{x+y}(i)$$ where $$GLCM_{x+y}(k) = \sum_{i=1}^{K}\sum_{j=1}^{K}\delta_{i+j,k} GLCM(i,j)$$ with k = 2, 3, …, 2K
Inverse difference momentum	$$Inv_{diffm} = \sum_{i=1}^{K}\sum_{j=1}^{K}\frac{1}{1+(i-j)^2} \cdot GLCM(i,j)$$

Variance (Haralic)	$Var_{xy} = \sum_{i=1}^{K}\sum_{j=1}^{K}(i-\mu_x)^2 \cdot GLCM(i,j) + \sum_{i=1}^{K}\sum_{j=1}^{K}(j-\mu_y)^2 \cdot GLCM(i,j)$<br>where<br>$\mu_x = \sum_{i=1}^{m}\sum_{j=1}^{m} iGLCM(i,j)$<br>$\mu_y = \sum_{i=1}^{m}\sum_{j=1}^{m} jGLCM(i,j)$
Entropy	$Entr = -\sum_{i=1}^{K}\sum_{j=1}^{K} GLCM(i,j) \cdot \log_2 GLCM(i,j)$
Sum entropy	$Sum_{entr} = -\sum_{i=2}^{2K} GLCM_{x+y}(i) \cdot \log_2 GLCM_{x+y}(i)$<br>where<br>$GLCM_{x+y}(k) = \sum_{i=1}^{K}\sum_{j=1}^{K} \delta_{i+j,k} GLCM(i,j)$<br>with k = 2, 3, …, 2K
Sum variance	$Sum_{var} = \sum_{r=2}^{2K}(i - Sum_{entr})^2 \cdot GLCM_{x+y}(r)$<br>where<br>$GLCM_{x+y}(k) = \sum_{i=1}^{K}\sum_{j=1}^{K} \delta_{i+j,k} GLCM(i,j)$<br>with k = 2, 3, …, 2K
Difference variance	$Diff_{var} = Var(GLCM_{x-y})$<br>where<br>$GLCM_{x-y}(k) = \sum_{i=1}^{K}\sum_{j=1}^{K} \delta_{\lvert i-j \rvert,k} GLCM(i,j)$<br>with k = 0, 1, …, K-1

Dissimilarity	$$Diss = \sum_{i=1}^{K}\sum_{j=1}^{K} GLCM(i,j) \cdot \lvert i-j \rvert$$
Information measure correlation 1	$$Inf_1 = \frac{HXY - HXY1}{\max\{HX, HY\}}$$ where $$HXY = -\sum_{i=1}^{K}\sum_{j=1}^{K} GLCM(i,j) \cdot \log(GLCM(i,j))$$ $$HXY1 = -\sum_{i=1}^{K}\sum_{j=1}^{K} GLCM(i,j) \cdot \log(GLCM_x(i)GLCM_y(j))$$ $$GLCM_x = \sum_{i=1}^{K} GLCM(i,j)$$ $$GLCM_y = \sum_{j=1}^{K} GLCM(i,j)$$ $$HX = -\sum_{i=1}^{K}\sum_{j=1}^{K} GLCM_x \cdot \log(GLCM_x)$$ $$HY = -\sum_{i=1}^{K}\sum_{j=1}^{K} GLCM_y \cdot \log(GLCM_y)$$
Information measure correlation 2	$$Inf_2 = \sqrt{(1 - \exp[-2 \cdot (HXY2 - HXY)])}$$ where $$HXY2 = -\sum_{i=1}^{K}\sum_{j=1}^{K} GLCM_x(i) \cdot GLCM_y(j) \cdot \log_2\{GLCM_x(i) \cdot GLCM_y(j)\}$$ $$GLCM_x = \sum_{i=1}^{K} GLCM(i,j)$$ $$GLCM_y = \sum_{j=1}^{K} GLCM(i,j)$$ $$HXY = -\sum_{i=1}^{K}\sum_{j=1}^{K} GLCM(i,j) \cdot \log(GLCM(i,j))$$
Inverse difference moment normalized	$$Inv_{diffm-norm} = \sum_{i=1}^{K}\sum_{j=1}^{K} \frac{GLCM(i,j)}{1 + \frac{(i-j)^2}{K}}$$

Standard deviation of GLCM	$\sigma_{GLCM} = \sqrt{\frac{1}{K \cdot K} \cdot \sum_{i=1}^{K} \sum_{j=1}^{K} \lvert GLCM(i,j) - \sum_{i=1}^{K} \sum_{j=1}^{K} \frac{GLCM(i,j)}{K \cdot K} \rvert^2}$
Variance of GLCM	$Var_{GLCM} = {\sigma_{GLCM}}^2$

Table S3 List of statistical features extracted from the GLRLM

We here list the statistical parameters extracted from the GLRLMs (Grey-Level Run-Length Matrices) calculated from the SP images. In the formulas reported in the following table, *K* represent the gray level (varying from 0 to 255); (*i*, *j*) represent the element with coordinates *i* and *j* in the GLRLM matrix.

Statistical feature	Mathematical expression
Short run emphasis	$SRE = \sum_{i=1}^{K} \sum_{j=1}^{M} \frac{GLRLM(i,j)}{j^2}$
Long run emphasis	$LRE = \sum_{i=1}^{K} \sum_{j=1}^{M} j^2 \cdot GLRLM(i,j)$
Gray level non uniformity	$GLN = \sum_{i=1}^{K} (\sum_{j=1}^{M} GLRLM(i,j))^2$
Run length non uniformity	$RLN = \sum_{j=1}^{M} \left(\sum_{i=1}^{K} GLRLM(i,j) \right)^2$

Low gray level run emphasis	$LGRE = \sum_{i=1}^{K}\sum_{j=1}^{M}\frac{GLRLM(i,j)}{i^2}$
High gray level run emphasis	$HGRE = \sum_{i=1}^{K}\sum_{j=1}^{M} i^2 \cdot GLRLM(i,j)$
Short run low gray level emphasis	$SRLGE = \sum_{i=1}^{K}\sum_{j=1}^{M}\frac{GLRLM(i,j)}{i^2 \cdot j^2}$
Short run high gray level emphasis	$SRHGE = \sum_{i=1}^{K}\sum_{j=1}^{M}\frac{GLRLM(i,j) \cdot i^2}{j^2}$
Long run low gray level emphasis	$LRLGE = \sum_{i=1}^{K}\sum_{j=1}^{M}\frac{GLRLM(i,j) \cdot j^2}{i^2}$
Long run high gray level emphasis	$LRHGE = \sum_{i=1}^{K}\sum_{j=1}^{M} GLRLM(i,j) \cdot i^2 \cdot j$

Table S4 Results of Tukey test

Tukey's HSD Pairwise Group Comparisons (99.0% Confidence Interval):

0 = OLIMEL N7E

1 = OLIMEL N12E

2 = OLIMEL N5E

3 = OLIMEL N4E

4 = FINOMEL

5 = NUMETA G13E

Comparison	Statistic	p-value	Lower CI	Upper CI
(0 - 1)	0.063	0.028	0.004	0.122
(0 - 2)	-0.394	0.000	-0.453	-0.334
(0 - 3)	0.379	0.000	0.319	0.438
(0 - 4)	1.078	0.000	1.020	1.137
(0 - 5)	2.305	0.000	2.247	2.364
(1 - 2)	-0.457	0.000	-0.516	-0.398
(1 - 3)	0.315	0.000	0.257	0.374
(1 - 4)	1.015	0.000	0.957	1.074
(1 - 5)	2.242	0.000	2.184	2.300
(2 - 3)	0.772	0.000	0.713	0.832
(2 - 4)	1.472	0.000	1.414	1.531
(2 - 5)	2.699	0.000	2.640	2.757
(3 - 4)	0.700	0.000	0.641	0.758
(3 - 5)	1.927	0.000	1.868	1.985
(4 - 5)	1.227	0.000	1.169	1.284

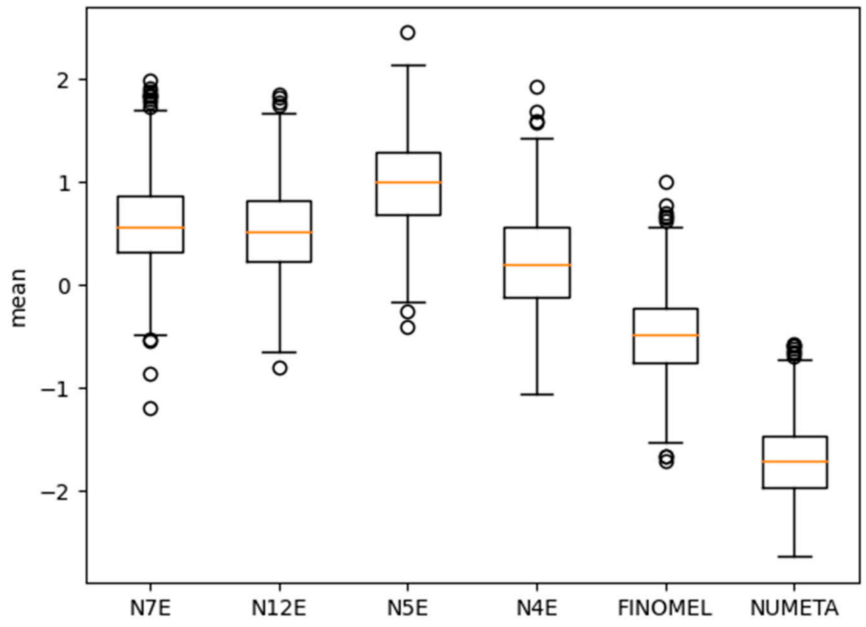

Figure S1. Boxplot representing the mean value of the gray-level intensity, the 25th and 75th percentile and the outliers for each PAN drug.

S.5 SHAP values

We here report the beeswarm plot of the SHAP values, extracted for every tested PAN drug, obtained for the MLP model.

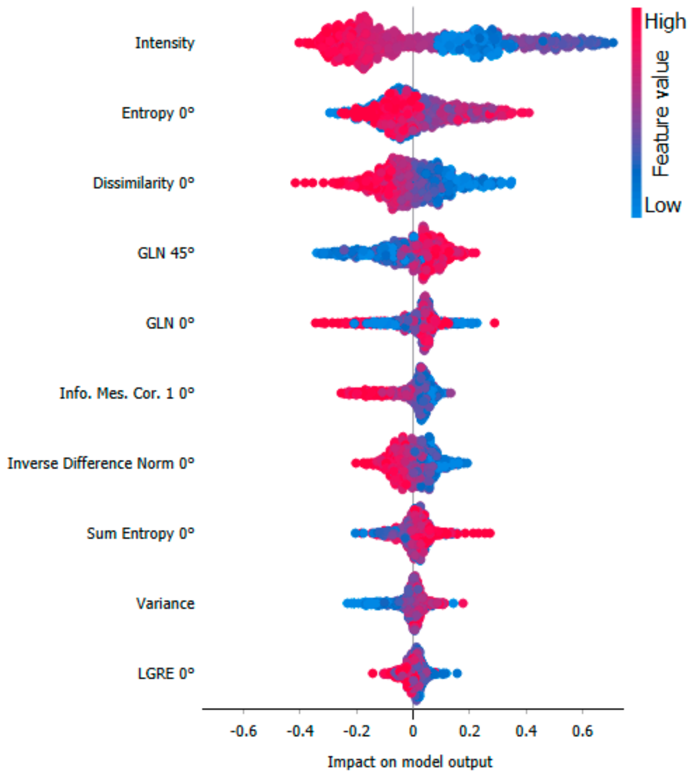

Figure S2. SHAP values related to FINOMEL (extracted after application of the MLP model).

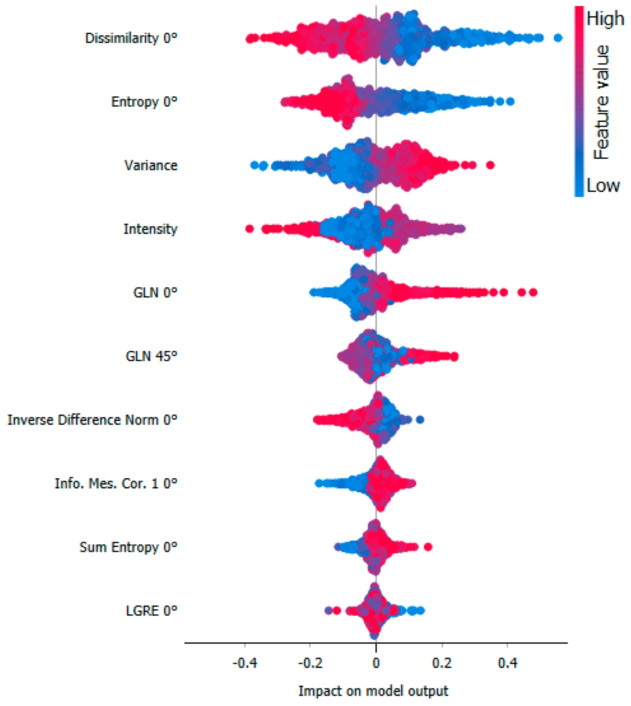

Figure S3. SHAP values related to OLIMEL N4E (extracted after application of the MLP model).

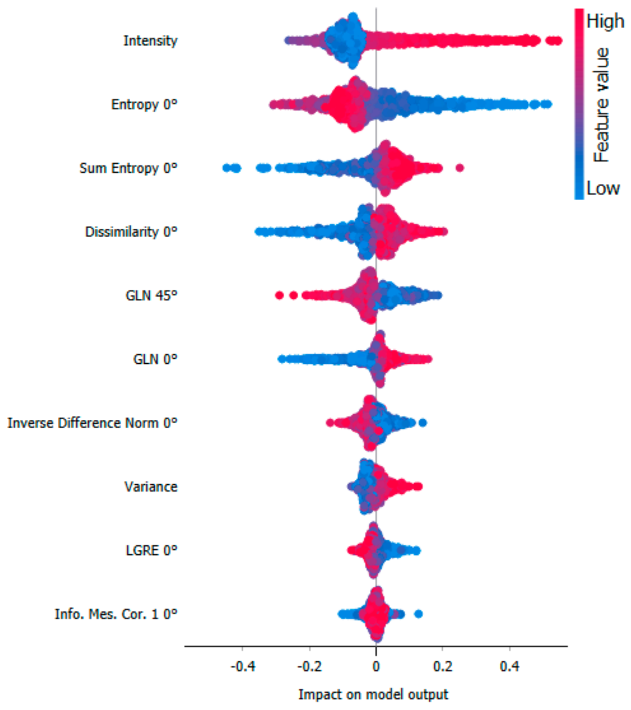

Figure S4. SHAP values related to OLIMEL N5E (extracted after application of the MLP model).

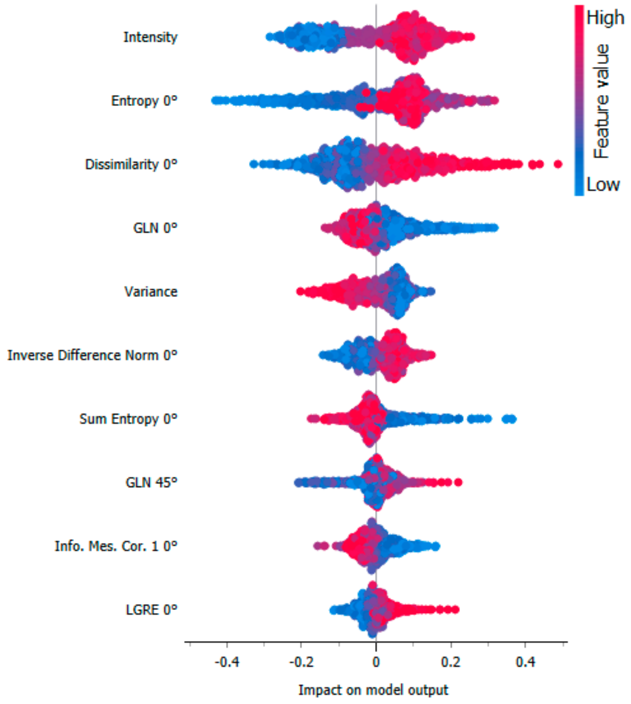

Figure S5. SHAP values related to OLIMEL N7E (extracted after application of the MLP model).

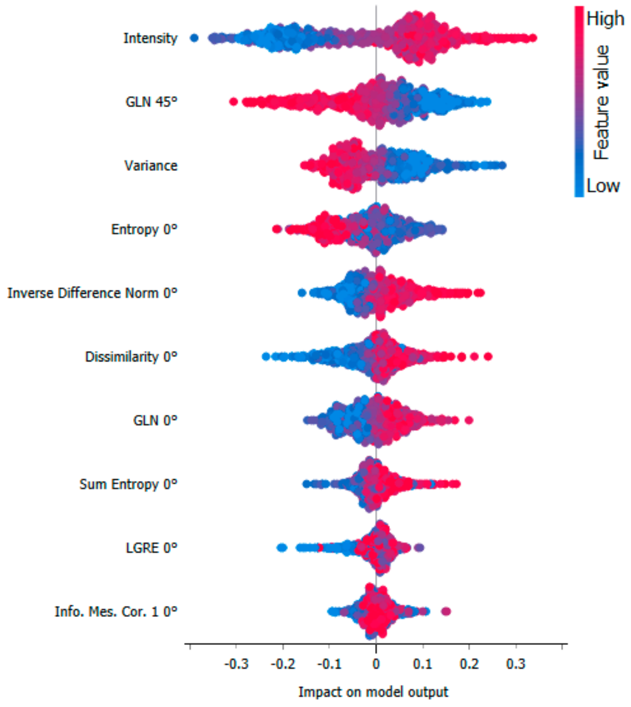

Figure S6. SHAP values related to OLIMEL N12E (extracted after application of the MLP model).

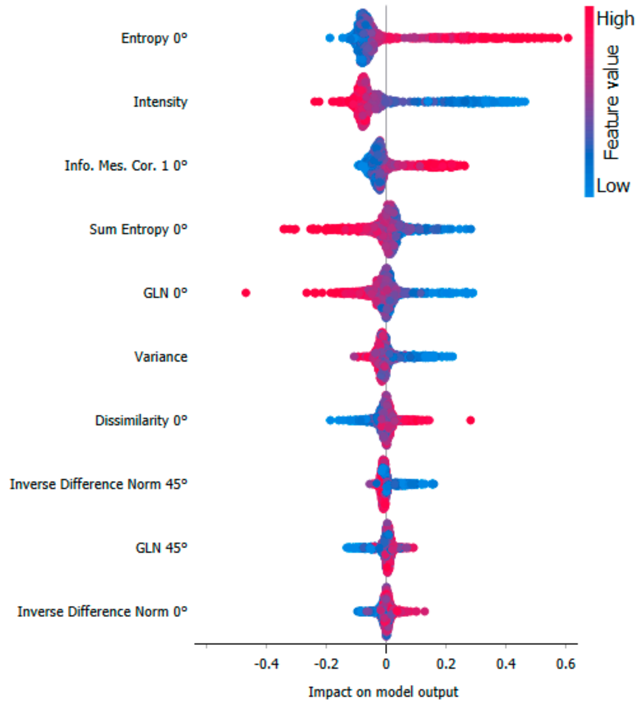

Figure S7. SHAP values related to NUMETA G13E (extracted after application of the MLP model).
